# Resistance and Resilience of Soil Nitrogen Cycling to Drought and Heat Stress in Rehabilitated Urban Soils

**DOI:** 10.3389/fmicb.2021.727468

**Published:** 2021-12-22

**Authors:** Mehdi Fikri, Catherine Joulian, Mikael Motelica-Heino, Marie-Paule Norini, Jennifer Hellal

**Affiliations:** ^1^BRGM, DEPA/GME, Orléans, France; ^2^ISTO, UMR 7327, CNRS-Université d’Orléans-Brgm, Orléans, France

**Keywords:** soil functions, resistance, resilience, N-cycling, bacteria, denitrification, nitrification, rehabilitation

## Abstract

In the context of climate change and biodiversity loss, rehabilitation of degraded urban soils is a means of limiting artificialization of terrestrial ecosystems and preventing further degradation of soils. Ecological rehabilitation approaches are available to reinitiate soil functions and enhance plant development. However, little is known about the long-term stability of rehabilitated soils in terms of soil functions when further natural or anthropogenic perturbations occur. Based on rehabilitated urban soils, the present study sought to evaluate the resistance and resilience of soil functions linked to carbon cycling and phosphate dynamics in addition to nitrogen cycling and related microbial communities after a heat and drought stress. A laboratory experiment was conducted in microcosms under controlled temperature conditions, with four contrasted soils collected from a rehabilitated urban brownfield; an initial, non-rehabilitated soil (IS), a technosol with a high organic matter level (HO), and two technosols with less organic matter (LO1 and LO2), together with their respective controls (no stress). Changes in potential denitrification (PDR), nitrification (PNR) rates, and their interactive relationships with soil microbial activities and soil physicochemical properties were determined following a combined heat (40°C) and drought stress period of 21 days. Measurements were carried out immediately after the stress (resistance), and then also 5, 30, and 92 days after soil rewetting at 60% water holding capacity (resilience). Microbial activities involved in soil functions such as carbon cycling and phosphate dynamics proved to be of low resistance in all soils except for IS; however, they were resilient and recovered rapidly after rewetting. On the other hand, the microbial activities and gene abundances that were measured in relation to nitrogen cycling processes showed that for denitrification, activities were more rapidly resilient than gene abundances whereas for nitrification the activities and gene abundances were resilient in the same way. Results suggest that, unless the soils contain high amounts of organic matter, microbial communities in imported soils can be more vulnerable to environmental pressures such as drought and heat than communities already present. This should be considered when rehabilitating degraded soils.

## Introduction

Anthropized soils have increased significantly in France since the first industrial revolution in the eighteenth century and the increasing urban sprawl. This has led to soil artificialization, which is defined as the process by which soils change use from an agricultural or natural state to a constructed building or a parking lot, for example (Pascual Aguilar et al., 2011), leading to the degradation of its ecological functions. Soil artificialization has occurred especially at the cost of agricultural lands [87% of artificialized soils in France between 2006 and 2015 used to be agricultural lands (CGDD, 2015)]. Additionally, brownfields with degraded soils can cover vast surfaces in urban and peri-urban areas. These are usually characterized by a low to moderate chemical contamination and limited fertility preventing their reuse without a prior rehabilitation process (Vincent et al., 2018). In light of the rising challenge to limit urban expansion at the expense of agricultural and natural terrestrial ecosystems, urban planning needs to take into consideration the requalification of brownfields and promote their productive potential. Moreover, the reuse of abandoned urban spaces and soil rehabilitation are key factors for protecting vital ecosystem services (Vincent et al., 2018).

Many studies have taken an interest in the rehabilitation of agricultural soils (De Noni and Viennot, 1993; Song et al., 2017; Nesper et al., 2019; Norton and Ouyang, 2019). However, to our knowledge, few studies have investigated soil ecological functions in rehabilitated urban soils. Lorenz and Lal (2009) reported that there are both similarities and differences concerning carbon (C) and nitrogen (N) biogeochemical cycling between urban and natural soils. These authors stated that the main differences reside in modifications of pH, compaction, moisture, and organic matter content in urban soils due to human activities, which influence critical soil biogeochemical processes like nitrification and denitrification. In addition, microbial communities in urban soils are substantially affected by human activities through the disturbance of biotic and abiotic soil properties (Li et al., 2018). Urbanization also affects nutrient cycling and their distribution, leading to increased N and P contents in soils (Sardans et al., 2006; Hobbie et al., 2017) as well as the accumulation of nutrients (Noe and Hupp, 2005). Moreover, urban environments may also suffer heat island effects, thus increasing soil temperature (Oke, 1989) which can significantly affect microbial activity (Carreiro et al., 2009) and affect major biogeochemical (C, N, and P) cycles in soils (Bapiri et al., 2010; Gleeson et al., 2010; Kaurin et al., 2018). Microorganisms are the main drivers for N-cycling due to their various adaptive strategies and their capacity to use various energy sources (Philippot et al., 2013). Nitrogen is an essential element for biota growth in soil (Jackson et al., 2008). N-cycling in terrestrial ecosystems is a complex process because N exists under gaseous, dissolved, and particulate forms. Nevertheless, in soils, N pathways are regulated by five interlinked microbial processes: fixation (N_2_ → NH_4_^+^), mineralization (N_org_ → NH_4_^+^), nitrate ammonification (NO_3_^–^ → NH_4_^+^), nitrification (NH_4_^+^ → NO_3_^–^), and denitrification (NO_3_^–^ → N_2_) (Hallin et al., 2009). Previous studies reported that soil’s physicochemical properties and abundances of related microbial genes are key factors in determining soil nitrification and denitrification (Jia and Conrad, 2009; Li et al., 2009; Hynes and Germida, 2012; Zhang et al., 2017; Zhang and Ji, 2018).

The increase in drought frequency and duration, which are typically combined with heat waves and interspersed rewetting, is raising questions concerning the capacity of microorganisms to adapt to such events in Mediterranean regions for example (Gibelin and Déqué, 2003; Guillot et al., 2019). Moreover, especially for agricultural soils, soil microbial communities can be resistant and even resilient to drought, as they are adapted to seasonal climatic extremes (Griffiths and Philippot, 2013; Kaurin et al., 2018). Therefore, in the context of soil rehabilitation it is essential to understand the response of N-cycling functions to drought periods, and the eventual impact on related microbial communities’ structure.

This article addresses knowledge gaps concerning rehabilitated urban soils in terms of the stability (resistance and resilience) of key microbial functions to heat and drought stress. The study investigates the effects of such a stress on global microbial activity and N-cycling functions (denitrification and nitrification) in rehabilitated urban soils. In addition, the effect of stress on specific microbial activities (basal respiration, phosphatase and β-glucosidase activities, and molecular microbial biomass) was also investigated. We hypothesized that soil’s N-cycling functions and microbial activities would be more resistant and resilient to heat (40°C) and drought stress in a soil with a high organic matter level. Finally, through this evaluation of the stability of several key soil microbial processes, this study also aims to compare three rehabilitation itineraries, two with low organic matter technosols with different plantations (LO1 and LO2) and an imported technosol with a high organic matter contents (HO), with the initial soil (IS) to give a first account of the best choice of rehabilitation.

## Materials and Methods

### Site Description and Soil Sampling

The experimental site of Pierre Bénite is located in Lyon metropolis in France. It has a semi-continental climate with an average annual temperature of 12°C and average annual rainfall of 1,015 mm. Summers are typically hot, and temperatures can reach 40°C. This site located along the Rhône riverbank was initially filled with alluvial sand and gravel then used for several industrial activities. A part of the brownfield was rehabilitated in 2016 during the REBU project (REBU, 2016), with the objective of developping vegetation cover and soil biota by restoring major biogeochemical cycles in soils (C, N, and P) and evaluating the best cost-effective approach. Several experimental modalities were set up using different imported or on-site materials and then received a mixture of PGPR (plant growth-promoting rhizobacteria) involved in nitrogen fixation and mycorrhiza combined with a seed composition adapted to the pedo-climatic conditions and containing a mixture rich in *Fabaceae* and *Poaceae*, as well as other species found on site. Selection of seed compositions and microbial inoculations were provided and carried out by Valorhiz (Montferrier-sur-Lez, France).^1^ In April 2019, 3 years after their rehabilitation, four of these modalities were chosen for the present experiment: the initial, non-rehabilitated soil consisting of a sandy material (IS modality); two modalities that had received the same imported low organic matter content technosol but with two different seed mixtures (LO1 and LO2 modalities, young plants and an accompanying seed composition and a “convalescent” seed composition, respectively); and an imported technosol rich in organic matter (HO) that received the same seed composition as LO1.

Surface soils (0–20 cm) were sampled nine times from each plot following an “M” pattern with an auger and put in sterile sampling bags (Whirl-Pak^®^). Composite samples were sieved at 4 mm in the laboratory and stored at 20°C for a few days until the experiment.

Soil textures were determined using a LaMotte 1067 soil texture kit (LaMotte Co., Chestertown, MD, United States), following the manufacturer’s protocol. IS is a loamy sand soil (80.7% sand, 16.2% silt, and 3.1% clay). LO1 (40% sand, 45.1% silt, and 14.7% clay) and LO2 (41.3% sand, 43.6% silt, and 15.1% clay) are loamy soils, and HO is a sandy loam soil (53.7% sand, 31.7% silt, and 14.8% clay).

### Experimental Setup

Microcosms consisted of 200 ml polystyrene pots filled with 100 g of equivalent dry soil and humidified to 60% water holding capacity (WHC), prepared in triplicates with soils from the four plots (IS, LO1, LO2, and HO, respectively). Half of the microcosms were controls, and the other half was used for stressing conditions. Before starting the experiment, all microcosms were pre-incubated for 2 weeks prior to the stress period to stabilize the microbial communities.

**FIGURE 1:**
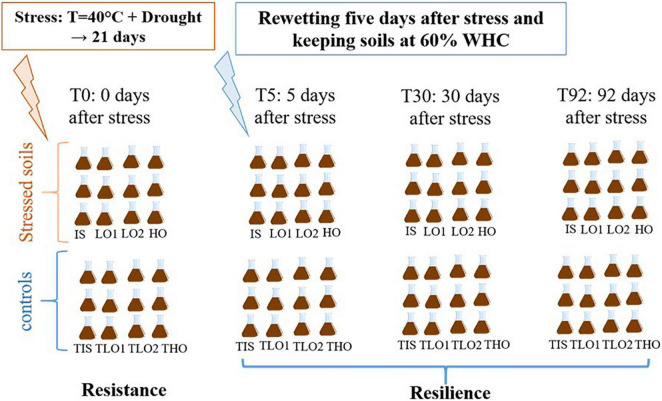
Experimental design conceptualized for the stress experiment for initial soil (IS), low organic matter soil 1 (LO1), low organic matter soil 2 (LO2), and high organic matter soil (HO).

Then, several steps were carried out as described in Figure 1.

**Step 1:** a heat (40°C) and drought stress period was applied to soils during 21 days; T0 corresponds to the end of this period for stressed soils and controls.

**Step 2:** the stressed soils were re-humidified to 60% WHC, and another sampling took place 5 days later (T5).

**Step 3:** Two more sampling operations were performed 30 days (T30), and 92 days after the stress ended (T92).

Controls and stressed soils from step 2 onward were incubated in a phytotron (plant growth chamber, Memmert HPP750 IPP PLUS, Büchenbach, Germany) at 20°C in the dark with 60% air–water saturation. Soil moisture was maintained at 60% WHC by weighing the soils once a week and, if necessary, adjusting the water contents with Mont Roucous mineral water (Ca^2+^ 2.9 mg⋅l^–1^; Mg^2+^ 0.5 mg⋅l^–1^; Na^+^ 3 mg⋅l^–1^; NO_3_^–^ 2 mg⋅l^–1^; SO_4_^2–^ 3 mg⋅l^–1^) of which the composition is comparable to rainwater. Basal respiration and phosphatase and β-glucosidase activities were measured at every sampling point, as described below. N-cycling functions were assessed by measuring potential denitrification rates (PDR) and potential nitrification rates (PNR), while N-cycling microbial communities were characterized by measuring gene abundances.

Soil samples for enzyme activities and microbial DNA extractions were frozen at –80 and –20°C, respectively. For N transformation rates, soil samples were dried at 60°C and kept in sealed containers for total N and total C measurements.

### Physicochemical Parameters

Soil pH_water_ was measured following the norm NF ISO 10390 (ISO, N. 10390, 2005). Total C, total H, and total N concentrations were measured in soil samples (5 mg of dry soil, ground and sieved at 160 μm to which ∼2 mg of vanadium pentoxide was added to accelerate the reaction) using an elemental Flash pyrolysis (Flash 2000, Thermo Fisher Scientific, Waltham, MA, United States). Total organic C was measured using the Rock-Eval pyrolysis technique (Rock-Eval 6 Turbo, Vinci Technologies, Nanterre, France).

### Soil Microbial Community Characterization

#### Microbial Basal Respiration

Microbial basal respiration was measured for all samples at every sampling time using the soil respiration system (MicroResp™ James Hutton Ltd., Aberdeen, United Kingdom, according to Campbell et al., 2003), following the manufacturer’s protocol.^2^ MicroResp™ is a colorimetric method based on CO_2_ release detection in a 96-well plate by a gel containing a colored indicator (cresol red) that changes color from purple to yellow when in contact with CO_2_. The CO_2_ emission by soil microorganisms was estimated before and after a 6 h incubation period at 25°C. The colorimetric assessment was performed at λ = 570 nm by an Omega SPECTROstar (BMG Labtech, Ortenberg, Germany) microplate spectrophotometer.

#### Enzymatic Activities

The activities of two microbial enzymes involved in C and P cycles were measured at each sampling time: ß-glucosidase (ß-Glu) which acts on the osidic bonds of glucose and degrades cellulose, and phosphatase (Phos) which degrades phosphatase esters and is responsible for catalyzing the phosphoester bond resulting in the liberation of phosphate. Measurements were carried out according to the norm (ISO, N. 20130, 2018), which uses a colorimetric method to detect the intensity of enzyme activities during an incubation period in microplates. Different substrates were used for the measurements of enzyme activities: 4-nitrophenyl β-D-glucopyranoside (CAS no. 2492-87-7) for ß-Glu quantification and 4-nitrophenylphosphate disodium salt hexahydrate (CAS no. 333338-18-4) for Phos quantification. Enzyme activity is measured by detecting the formation of para-nitrophenol (PNP) which was read at λ = 405 nm using an Omega SPECTROstar (BMG Labtech) microplate spectrophotometer. Results were expressed in mU.g^–1^_dry soil_ (namely, nmol⋅min^–1^⋅g^–1^_dry soil_).

#### Soil DNA Extraction and Quantitative Real-Time PCR

Soil DNA extractions were carried out at each sampling time on subsamples of between 0.4 and 0.8 g dry soil with the FastDNA^®^ Spin Kit for soil and the FastPrep-24™ instrument following the manufacturer’s (MP Biomedicals, Santa Ana, CA, United States) protocol and the following specific conditions: samples were homogenized for 30 s at a speed setting of 5.0 m⋅s^–1^, then centrifuged for 20 min at 14,000 rpm. DNA was quantified using the Quantus™ fluorometer (Promega, Charbonnières-les-Bains, France) with the Promega QuantiFluor^®^ dsDNA System, following the manufacturer’s recommendation.

Quantitative real-time PCR (qPCR) was used to measure bacterial abundance in soils by quantifying 16S rRNA gene copies. All samples were run in duplicates in a CFX96 Optical Real-Time detection System (Bio-Rad Laboratories, Inc., Hercules, CA, United States) in a 20 μl final volume containing 10 μl of SsoAdvanced Supermix (Bio-Rad), 0.16 μl of forward primer 341F (5′-CCTACGGGAGGCAGCAG-3′) (50 μM), 0.16 μl reserve primer 515R (5′-ATTACCGCGGCTGCTGGCA-3′) (50 μM), 0.2 μl T4 GP 32 (500 ng⋅μl^–1^; MP Biomedicals), 2 μl DNA at 1 ng⋅μl^–1^, and ultrapure water. Thermocycling conditions were as follows: 3 min at 95°C; 35 cycles of 30 s at 95°C, 30 s at 60°C, 30 s at 72°C, and 30 s at 80°C. N-cycling’s functional gene abundances were also measured using qPCR in a CFX96 system, in a 20-μl final volume with 10 μl of SsoAdvanced Supermix (Bio-Rad), 0.2 μl of each primer (50 μM) (Table 1), 0.2 μl T4 GP32 (500 ng⋅μl^–1^) (only for the *narG* gene), and 2 μl of DNA at 1 ng⋅μl^–1^, completed with ultrapure water. Thermal cycling conditions were as follows: for the *nosZ* gene: 2 min at 95°C; 40 cycles of 15 s at 95°C, 15 s at 62°C, 30 s at 72°C, and 30 s at 80°C; for the *narG* gene: 3 min at 95°C; 6 cycles consisting of 30 s at 95°C and 30 s at 63°C with a touchdown at –1°C by cycle and 30 s at 72°C; and 34 cycles consisting of 30 s at 95°C, 30 s at 58°C, 30 s at 72°C, and 30 s at 80°C; and for the *amoA* gene: 5 min at 95°C; 40 cycles consisting of 15 s at 95°C, 30 s at 55°C, and 30 s at 72°C, and 30 s at 80°C. At the end of each qPCR, a melting curve analysis was generated by applying a final step of 0.5°C temperature increment every 10 s, from 65 to 95°C.

**TABLE 1:** Primers used for the determination of functional genes related to N-cycling in soils.

Genes	Primers	Target	Base sequences	References
*nar*G	*narG*-F	Nitrate reductase	TCGCCSATYCCGGCSATGTC	Bru et al., 2007
	*narG*-R		GAGTTGTACCAGTCRGCSGAYTCSG	
*nos*Z	*nosZ*-2F	Nitrous oxide reductase	CGCRACGGCAASAAGGTSMSSGT	Henry et al., 2006
	*nosZ*-2R		CAKRTGCAKSGCRTGGAGAA	
*amo*A	*amoA*-1F	Bacterial ammonia mono-oxygenase	GGGGTTTCTACTGGTGGT	Rotthauwe et al., 1997
	*amoA*-2R		CCCCTCKGSAAAGCCTTCTTC	

For each gene, calibration curves were obtained using serial dilutions of a known quantity of linearized plasmids containing known copy numbers of the gene. Blank controls without DNA with ultrapure water were carried out in every qPCR analysis. The amplification efficiencies of target genes ranged between 87.3 and 96.3%, with *R*^2^ values between 0.99 and 1.

#### Determination of Soil N Transformation Rates

PDR were determined by the acetylene block technique of Petersen et al. (2012) and later used by Zhang et al. (2018). The method consists of measuring N_2_O emissions by gas chromatography (TRACE™ 13,000) after adding acetylene to block its transformation to N_2_ in anaerobic conditions at the end of a 24 h incubation period.

PNR were determined by placing soils (5 g equivalent dry soil) into 70 ml containers. Soils were further treated with 100 mgN⋅kg^–1^ (dry weight) (NH_4_)_2_SO_4_. Samples were adjusted to 60% WHC using deionized water and were incubated at 20°C for 7 days. At the end of the incubation period, samples were extracted with a 2 M KCl solution (5:1 solution of soil ratio). Extracts were shaken vertically at 200 rpm for 1 h, then later filtered with 0.2 μm acetate cellulose ClearLine^®^ filters. Extracts were analyzed for N-NH_4_, N-NO_2_, and N-NO_3_ concentrations using colorimetric techniques, Thermo Fisher Gallery™ for ammonium (references 984362 and 984363) and nitrite (reference 984371), and a Merck kit adapted for high salinity because of the extractant used (KCl) (Reference 1.14942.0001). Results were expressed in mgN⋅day^–1^⋅kg^–1^_dry soil_ between day 0 and day 7.

#### Statistical Analysis

Kruskal–Wallis multiple comparison tests were conducted to compare mean values at the 5% level using R software R-3.6.1 (R Core Team, 2018), agricolae package. Differences were considered significant for *p*-values <0.05. Data of all soils at all measurement periods were analyzed by principal component analysis also using R software, FactoMineR and factoextra packages.

## Results

### Initial Soil Physicochemical Properties

All soil pH ranged between 7.6 and 9.1, and IS had the highest pH value, followed by HO, LO2, and LO1 (Table 2). HO and IS had the highest total C contents followed by LO1 and then LO2. HO’s TOC content was significantly higher (∼7-fold) than all other soils, followed by LO1, then LO2 and finally IS. Total N contents ranged between 0.5 and 1.6 g⋅kg^–1^_dry soil_ for all soils, and LO1 and HO had the highest values, followed by LO2, then IS. HO’s C/N was significantly higher (∼3-fold) than all other soils, followed by LO2 and LO1, then IS. HO’s H content was significantly higher (∼2-fold) than all other soils, followed by LO1, then LO2 and finally IS. HO and LO2 N-NH_4_ contents were significantly higher (∼5-fold the minimal value) than LO1 and IS. N-NO_3_ contents ranged between 6.9 mgN⋅kg^–1^ and 17.7 (mgN⋅kg^–1^); LO1 had the highest value, followed by HO, LO2, and IS.

**TABLE 2:** Physicochemical properties of soils at T0.

Samples	pH	Total C (g⋅kg^–1^)	TOC (g⋅kg^–1^)	Total N (g⋅kg^–1^)	C/N	Total H (g⋅kg^–1^)	N-NH_4_ (mg_N–NH4_⋅kg^–1^)	N-NO_3_ (mg_N–NO3_⋅kg^–1^)
IS	9.1±0.0a	3±0.0a	3.7±0.5d	0.6±0.0c	6.3±0.1ab	1.8±0.1d	5±0.8b	6.9±0.1b
LO1	7.6±0.1d	1.1±0.0b	8.4±0.1b	1.6±0.1a	5.2±0.3b	3.5±0.1b	5.4±0.9b	17.7±0.1a
LO2	7.8±0.0c	0.95±0.3c	7.7±0.2c	1.3±0.2b	5.7±0.7b	3±0.3c	29±5a	10.4±0.3ab
HO	8±0.0b	3±0.3a	25±3a	1.5±0.2a	16.1±1.7a	3.9±0.2a	25±3.5a	14±0.2a

*Sample names refer as follows: initial soil (IS), low organic matter (LO1), low organic matter 2 (LO2), high organic matter (HO). Mean values and error of means are indicated; C, N, and H measurements were carried out for six replicates while other parameters are in triplicates. Significant differences found using the Kruskal–Wallis test are indicated with different letters.*

### Characterization of Soil Microbial Communities and Activities

#### Changes in Total Microbial Biomass and Enzymatic Activities

**FIGURE 2:**
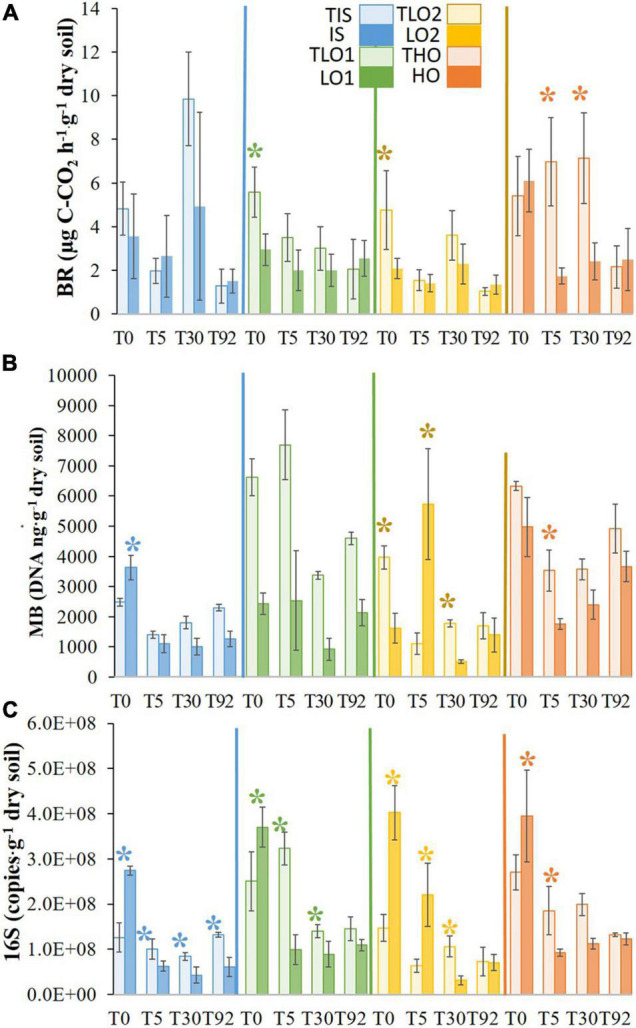
Means of basal respiration **(A)**, molecular biomass **(B)**, and bacterial abundance **(C)** for initial soil (IS), low organic matter soil 1 (LO1), low organic matter soil 2 (LO2), and high organic matter soil (HO), and their controls TIS, TLO1, THO, and TLO2 at different sampling times (T0, T5, T30, and T92). Statistical tests were performed using the Kruskal–Wallis test. The asterisks indicate a significant difference (*p*< 0.05) between a stressed soil and its control at the same sampling time. Error bars represent the error of means.

Basal respiration (BR) was measured at all sampling times to assess the level of microbial activity in the microcosms (Figure 2A). Just after the stress at T0, BRs of LO1 and LO2 were significantly lower than those of controls TLO1 and TLO2 (∼2- and 2.5-fold, respectively). In contrast, BRs in soils HO and IS (6.1 and 3.6 μgC-CO_2_⋅h^–1^⋅g^–1^_dry soil_, respectively) were not affected by the stress as no significant differences were found compared to BRs of controls THO (5.4 and 4.8 μgC-CO_2_⋅h^–1^⋅g^–1^_dry soil_, respectively). After rewetting at T5, only the BR of soil HO was found to be significantly lower than that of control THO (∼4-fold), while no significant differences were found for all the other soils. At T30, HO’s BR was the only measurement that was significantly lower than its control THO (∼3-fold). No significant differences were found between treatments and controls at T92. However, we observed that overall BR declined between T0 and T92 for all unstressed soils.

The heat and drought stress significantly decreased the molecular biomass (MB) of LO1 and LO2 soils compared to controls at T0 (∼3- and 2.5-fold, respectively) (Figure 2B). In contrast, MB in HO was not significantly affected, and MB in IS increased significantly (∼1.5-fold) compared to controls at T0. Five days after rewetting, MB in LO2 had increased significantly (∼5-fold) compared to control. In contrast, IS, LO1, and HO’s MB were significantly lower (∼1.3-, 3-, and 3-fold) than controls at T5. MBs in LO1 and LO2 were significantly lower than those of controls (∼4- and 3.5-fold) at T30; in contrast, no significant differences were found between HO and its control. Finally, at T92, no significant differences were found between stressed soils and controls. The stresses also significantly increased bacterial abundance (16S rRNA gene copies) in soils compared to controls at T0 (Figure 2C). However, 5 days (T5) after rewetting the soils, bacterial abundance decreased significantly for the IS, LO1, and HO soils (∼4-fold) compared to controls and LO2 was less affected (∼2-fold decrease). At T30, 16S rRNA gene abundance was still significantly lower in stressed soils than in the controls, except for HO. At the final sampling time T92, 16S rRNA gene abundance was approximately the same as controls in all soils except for IS where it was still significantly lower. Finally, results showed that 16S rRNA gene abundance had a tendency to decrease with the incubation time, as abundances were significantly lower at T92 compared to T0 for all soils.

**FIGURE 3:**
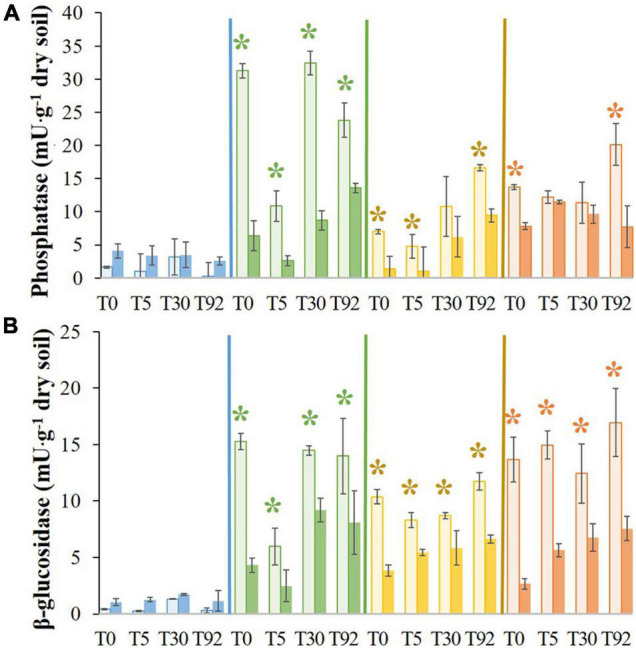
Means of phosphatase activity **(A)** and β-glucosidase **(B)**, for initial soil (IS), low organic matter soil 1 (LO1), low organic matter soil 2 (LO2), and high organic matter soil (HO), and their controls TIS, TLO1, THO, and TLO2 at different sampling times (T0, T5, T30, and T92). Statistical tests were performed using the Kruskal–Wallis test. The asterisks indicate a significant difference (*p*< 0.05) between a stressed soil and its control at the same sampling time. Error bars represent the error of means.

After the stress at T0, phosphatase activities in LO1, HO, and LO2 decreased significantly (∼5-, ∼2-, and ∼5-fold, respectively) compared to the controls TLO1, THO, and TLO2 (Figure 3A). On the contrary, activity increased significantly (∼2.5-fold) compared to its control TIS. At T5, 5 days after rewetting the stressed soils, phosphatase activities in LO1 and LO2 remained significantly lower (∼4- and ∼5-fold, respectively) than controls TLO1 and TLO2. On the contrary, no significant differences were found between IS and HO and their controls TIS and THO at T5. Furthermore, only one significant difference was found at T30 which was LO1 being lower than TLO1 (∼4-fold). In all stressed soils at T92, phosphatase activities were significantly lower than in controls at T92 except for IS. IS showed the lowest activity compared to other soils.

Results presented in Figure 3B show that at T0, β-glucosidase activities in LO1, LO2, and HO were significantly lower (∼3.5-, ∼3-, and ∼5-fold, respectively) than the controls TLO1, TLO2, and THO. Then, the β-glucosidase activity in these soils remained significantly lower than in controls all along the experiment at T5 (∼2.4-, 1.5-, and 2.6-fold, respectively), T30 (∼6-, 1.5-, and 2-fold, respectively), and T92 (1.1-, 1.7-, and 2.2-fold, respectively). In contrast, no significant differences were found for IS between controls and treatments after stress, although its activity was the lowest compared to other soils.

#### Changes in Denitrification and Nitrification Functions

**FIGURE 4:**
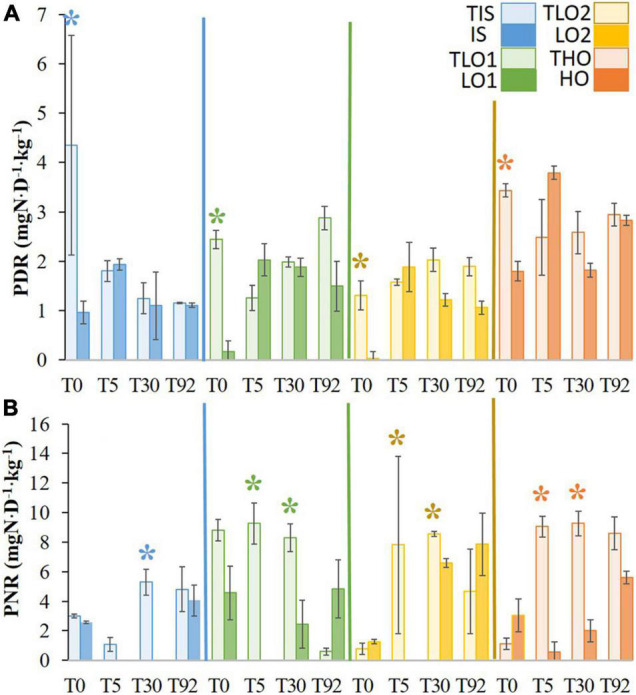
Means of potential denitrification rates (PDR) **(A)** and potential nitrification rates (PNR) **(B)** for initial soil (IS), two soils with less organic matter (LO1 and LO2), and high organic matter soil (HO) and their respective controls (TIS, TLO1, TLO2, and THO). Statistical tests were performed using the Kruskal–Wallis test. The asterisks indicate a significant difference (*p*< 0.05) between a stressed soil and its control at the same sampling time. Error bars represent the error of means.

After the stress, PDR decreased significantly in all soils compared to the respective controls (Figure 4A). Five days after the stress and rewetting (T5), all previously affected soils recovered PDR at the same order of magnitude as their respective controls. Furthermore, no significant differences in PDR were found between soils and controls at T30 and T92 either.

At T0 just after the stress, no significant differences in PNR were found between treatments and controls (Figure 4B). After rewetting (T5), PNR decreased significantly for all stressed soils compared to controls except for IS. PNR were significantly lower in stressed soils, compared to controls at T30 for all soils. At T92, no significant differences in PNR were found between stressed soils and controls.

#### Changes in Denitrifying and Nitrifying Microbial Communities

**FIGURE 5:**
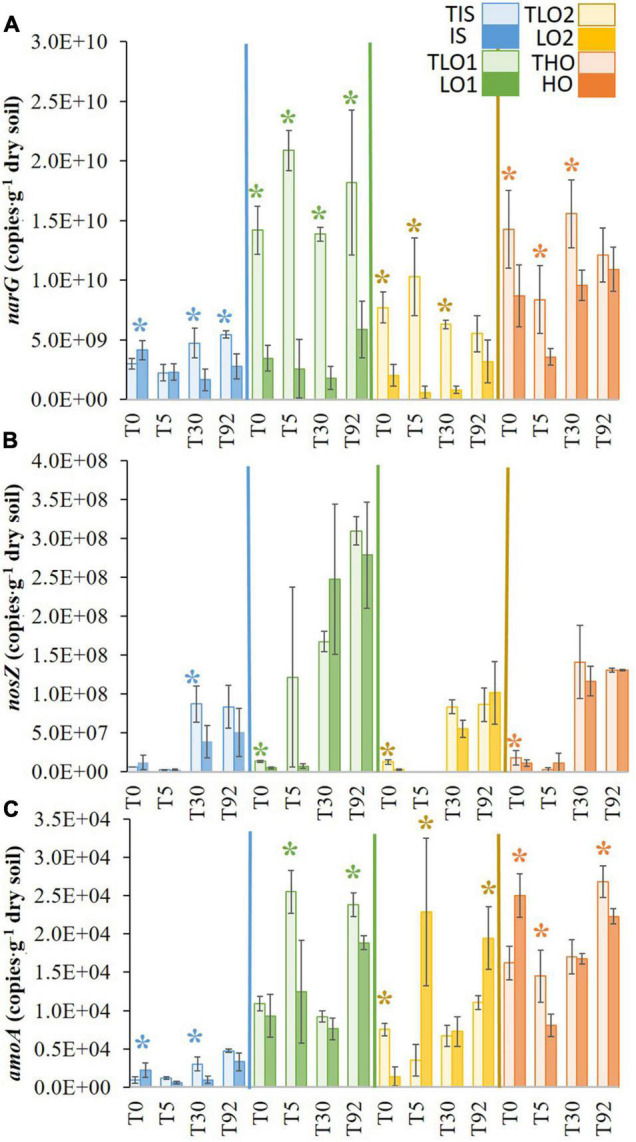
Means of *narG* **(A)**, *nosZ* **(B)** and *amoA* **(C)** gene abundances in the soils at different sampling times for initial soil (IS), high organic matter soil (HO), and two soils with less organic matter (LO1 and LO2) and their respective controls (TIS, THO, TLO1, and TLO2). Statistical tests were performed using Kruskal–Wallis. The stars indicate a significant difference (*p*< 0.05) between soil and control at the same sampling time. Error bars represent the error of means.

At T0, the stress significantly decreased the *narG* gene copy abundances in LO1, LO2, and HO soils (∼4-, 7-, and 1.6-fold, respectively) compared to controls TLO1, TLO2, and THO (Figure 5A). In contrast, the stress significantly increased the abundance of *narG* gene copies in IS at T0 (∼1.3-fold) compared to control TIS. Five days after rewetting at T5, *narG* gene abundances for all soils were significantly lower than controls except for IS. Thirty days after the stress period (T30), *narG* gene abundances were significantly lower than controls in all soils. At the final sampling time T92, *narG* gene copy numbers in IS and LO1 were significantly lower (∼2- and 3-fold, respectively) than controls TIS and TLO1. In contrast, no significant differences were found between HO, LO2, and their controls THO and TLO2 at T92.

Although *nosZ* gene copy abundances were low in all soils at T0, significant differences were found between the stressed soils and their controls for LO1, HO, and LO2 (∼2.6-, 1.6-, and 4.6-fold, respectively) compared to controls TLO1, THO, and TLO2 (Figure 5B). Five days after rewetting (T5), no significant differences were found between soils and controls. Thirty days after the stress (T30), *nosZ* gene abundance in IS was significantly lower (∼2-fold) than the control TIS, whereas no significant differences were found between all other soils and controls at T30. At the final sampling time T92, no significant differences were found between treatments and controls for all soils. The results showed that *nosZ* gene abundances were significantly higher at T30 and T92 compared to T0 and T5 and that the number of *nosZ* gene copies increased significantly after T5 for all soils and controls.

After the stress period at T0, both IS and HO had significantly higher (∼2- and 1.5-fold, respectively) *amoA* gene abundances than controls TIS and THO. No significant difference was found between TLO1 and LO1, while LO2 was significantly lower (∼2-fold) than TLO1 (Figure 5C). Five days after rewetting (T5), *amoA* gene abundances were significantly higher compared to controls for LO2 (∼6-fold) whereas they were lower for LO1 and HO (∼2- and 1.8-fold, respectively) and not significantly different for IS. Thirty days after the stress (T30), *amoA* gene abundances were significantly lower (∼3-fold) than controls in IS, whereas no significant differences were observed for the other soils. At the final sampling date (T92), *amoA* gene abundances were significantly higher than controls for LO2 (∼2-fold) whereas they were comparable to controls for for LO1, HO and IS.

### Principal Component Analysis of Biological Parameters

**FIGURE 6:**
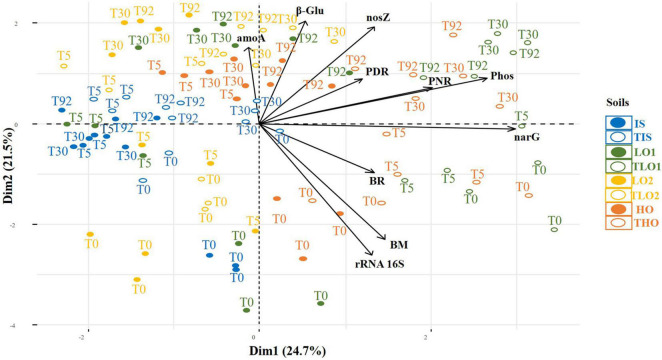
Principal component analysis (PCA) of soil initial soil (IS), two soils with less organic matter (LO1 and LO2), and high organic matter soil (HO), and their respective controls (TIS, THO, TLO1, and TLO2), at different sampling times (T0, T5, T3, and T92). The data analyzed are PNR (potential nitrification rates), PDR (potential denitrification rates), BR (basal respiration), phosphatase and β-glucosidase activities, MB (molecular biomass), phosphatase and *nosZ*, *narG*, and *amoA* gene abundances.

PCA was carried out on the following parameters: PDR, PNR, phosphatase activity (Phos), β-glucosidase activity (β-Glu), BR, BM, and *nosZ*, *narG*, *amoA*, and 16S rRNA gene abundances for all soils at all sampling times. Results indicated that PC1 and PC2 accounted for 43.8% of the total variance (Figure 6). THO and TLO2 soils were positioned on the right side of PC1, mainly explained by *narG* gene abundances, Phos and PNR. The PCA also showed that the positions of soils and controls at T0 tended to be in the lower part of the plot along the second axis, whereas soils and controls from other sampling times (T5, T30, and T92) were positioned higher up along the second axis, which is mainly driven by 16S rRNA gene abundances, MB and β-Glu (Figure 6). No clear correlations between PNR, PDR, and corresponding *amoA* gene abundances and *nosZ* and *narG* gene abundances could be distinguished from the PCA. Furthermore, linear regressions (data not shown) did not demonstrate any significant correlations either between PDRs and functional gene abundances (*narG* and *nosZ*), or between PNR and *amoA* gene abundances.

## Discussion

### Heat and Drought Stress Affected Carbon Mineralization and Nutrient Dynamics on the Short Term

#### Carbon Mineralization and Phosphate Dynamics Were Not Resistant to Stress

The microbial process of carbon mineralization, which contributes to the ecological function of organic matter stock and dynamics, was evaluated by measuring microbial MB, bacterial abundance, soil respiration, and β-glucosidase activity and generally decreased following heat and drought stress. Microbial biomass was measured using MB, the quantity of DNA extracted from soil which is an indicator of the biological state of an environment (Dequiedt et al., 2011; Bouchez et al., 2016) and was durably impacted by stress in the present study. BR was the most affected in LO1 and LO2 after the stress period (T0). Previous work made similar observations where heat and drought stress drastically affected BR in French agricultural soils (Bérard et al., 2012), in tropical soils, and in Australian agricultural soils (Liang et al., 2014; Yu et al., 2014, respectively). The reasons behind this decrease may be a physiological effect on microorganisms, as drought can cause cell desiccation and lysis (Göransson et al., 2013), or the thermal denaturation of microbial enzymes (Bérard et al., 2011). However, bacterial abundance appeared to increase in all soils directly after stress (T0) which was in contradiction with the other results. β-Glucosidase is an enzyme responsible for the breakdown of several forms of carbohydrates, especially cellulose, which make its role fundamental for nutrient availability and cycling (Singhania et al., 2013). Compared to their respective controls, β-glucosidase activity (β-glu) decreased significantly after the stress (T0) in all soils except IS, indicating a low resistance of this enzymatic activity to heat and drought stress. Our results are comparable to other authors who have also reported a decrease in soil β-glu due to drought stress in Mediterranean grasslands and subtropical acrisol (Sardans and Peñuelas, 2005; Liang et al., 2014, respectively), grass plantations (Sanaullah et al., 2011), and Mediterranean semiarid soils (Hueso et al., 2011). Heat and drought stress can induce a critical situation for soil microbial communities causing cell death, and thus an alteration of enzymatic activities involved in C-cycling (Lamersdorf et al., 1998).

Phosphatase activity is involved in the transformation of phosphorous compounds (Amador et al., 1997), and its activity is essential in P-cycling. Its activity contributes to the process of phosphate cycling and the ecological function of nutrient availability to plants and soil organisms. Phosphatase activities were significantly lower in all soils at T0 in the stressed soils compared to the controls except in IS, showing a low resistance of the activity to drought and heat stress. These findings are in accordance with studies showing that experimental drought stress reduced alkaline phosphatase activity in *Quercus ilex* Mediterranean forest soils (Sardans et al., 2008) and in Mediterranean terrestrial ecosystems (Sardans and Peñuelas, 2005).

Thus, heat and drought stress impacted microbial functions involved in carbon mineralization and phosphate dynamics in rehabilitated urban soils, demonstrating the vulnerability of these soils.

#### Carbon Mineralization and Phosphate Dynamics’ Resilience to Stress

The following sampling times, T5, T30, and T92 after soil rewetting, enabled to monitor the bacterial communities’ resilience to the heat and drought stress for the different ecological functions. Overall, except for MB in LO1, carbon mineralization was resilient for MB, BR, and bacterial abundance after 92 days, as was found by Bérard et al. (2012), in which BR recovered after 96 days. Microorganisms have evolved defense mechanisms allowing them to recover after perturbation. Some heat-tolerant bacteria like *Actinobacteria* can survive desiccation stress by increasing synthesized ribosomes to grow quickly once conditions become favorable (Barnard et al., 2013). This could have been the case for our soils as microorganisms could have adopted this preparation strategy, accelerated their cell division, and increased their biomass after re-humidification. The *Firmicutes* phylum also includes bacteria with heat and drought tolerance mechanisms (De Vos et al., 2009). Additionally, if bacteria from phyla such as *Firmicutes* and *Actinobacteria* were favored during the stress period, this may explain the increase in the copy numbers of the gene coding for 16s rRNA as both these phyla have been shown to possess multiple copies of this gene in their genome (Sun et al., 2013; Větrovský and Baldrian, 2013).

However, the heat and drought stress irreversibly altered β-glu and phosphatase activities. Previous studies have reported that drought can strongly limit enzymatic activities (Henry, 2013; Siebielec et al., 2020). In a previous study involving a heat stress (50°C), β-glu activity decreased by twofold in French agricultural soils (Riah-Anglet et al., 2015), which is somewhat comparable with HO where the activity decreased by 1.7-fold. Drought also reduced phosphatase activity in forest soils in Spain (Sardans and Peñuelas, 2010). This could be explained by the fact that heat stress can cause a decrease in enzyme synthesis and secretion (Allison, 2005). Our results showed that with the exception of IS, none of the soil β-glu and phosphatase activities had recovered after 92 days. Siebielec et al. (2020) found more contrasting results as phosphatase activity had a full recovery in loamy agricultural soils after a 1-month drought period.

Although microbial functions involved in carbon cycling and phosphate dynamics were poorly resistant to the drought and temperature stress, they appeared to recover rapidly as discussed. However, future work could question how many stress events such soils could support before irreversibly affecting these functions, in turn altering nutrient availability and disrupting the cycling of essential elements.

#### Organic Matter Contents in the Rehabilitated Soils and Acclimation in Existing Soils Could Explain the Resilience of Carbon Mineralization

IS was the initial, un-rehabilitated soil and did not undergo any modifications during the initial REBU project. Therefore, although overall biomass and activities were lower in this soil compared to the others, its intrinsic microbial communities could be more adapted to drought and heat conditions by regularly undergoing seasonal changes. Similar findings were reported in a study where heat and drought stress (21-day period) had less impact on the BR of soils that were pre-exposed to stress, compared to those which were not pre-exposed in French agricultural soils (Bérard et al., 2012). Furthermore, it has already been evidenced that for soil microbial communities, a preselection by drying and rewetting reduces the impact of additional drought stress (Fierer et al., 2003; Williams, 2007; Bouskil et al., 2013; Zeglin et al., 2013; Wang et al., 2014; Bérard et al., 2015). This is probably because drought causes changes in the ecophysiology of microbial communities, and a pre-exposure to stress favored a fraction of microbial communities best adapted to heat and drought conditions (Anderson and Domsch, 1985, 1993).

Soil HO showed the highest total organic carbon content (Table 2). On the contrary to LO1 and LO2, HO’s BR was not affected by heat and drought stress. This might be explained by its higher content of available C making microbial communities more resistant to stress (Sanaullah et al., 2011; Canarini et al., 2017). It is known that organic C is an important driver of BR in soils (Romero-Freire et al., 2016), and drought affects soil processes indirectly by altering the substrate’s availability to microorganisms (Schimel, 2018). This can be explained by the decline of diffusion rates when drought increases, as microorganisms become resource deprived which limits their capacity of using acclimation methods that require carbon and energy (Schimel et al., 2007). This suggestion is further supported by the fact that HO’s MB was resistant to drought, in contrast to LO1 and LO2, which declined. This can be explained by microorganisms resorting to dormancy to avoid stress, therefore reducing growth efficiency and allocating more resources to survival instead of reproduction (Schimel et al., 2007). Higher organic matter content in soil can improve water retention, therefore mitigating water availability for microorganisms. In a study conducted in US maize fields, results showed that higher organic matter content in soils was associated with higher crop yields. Analyses indicated that this positive association of soil organic matter and yields is partially explained by positive effects of soil OM on available water capacity (Kane et al., 2021). This is crucial for microbial survival as water loss can cause a loss of cell turgor (Harris, 2015), which can be catastrophic for microorganisms and result in interferences with physiological functions, reduce metabolic processes, and eventually lead to cell death (Schimel, 2018).

In turn, low organic matter contents could affect other soil microbial processes such as phosphatase activity and nutrient availability (Bonmati et al., 1991; Olander and Vitousek, 2000), which could explain the overall low values of phosphatase activity in IS, correlating with the low N and TOC values, whereas HO’s phosphatase activity was resilient. IS also had the lowest β-glu activity compared to other soils, which might be due to less available organic substrates for β-glu such as cellulose (Hueso et al., 2011). This finding is in accordance with other studies where the increase in organic carbon enhanced soil β-glu activity (Eivazi and Tabatabai, 1990; Piotrowska and Koper, 2010).

### Nitrogen Cycling Processes Denitrification and Nitrification Were Affected Differently by Drought and Heat Stress

#### Denitrification Activities Were More Rapidly Resilient Than Gene Abundance

The effects of drought and heat stress on the nitrogen cycle were evaluated by determining both the nitrification and denitrification potentials in soils and the abundances of functional genes involved in soil N-cycling. All stressed soils showed low resistance for PDRs to drought and heat stress. Many studies have shown that drought decreases the denitrification process in different terrestrial ecosystems like tropical forests (Almaraz et al., 2019), grasslands (Hartmann et al., 2013; Keil et al., 2015), and agricultural fields (Homyak et al., 2017). However, even though they were impacted by the stress, PDR in all soils recovered quickly after rewetting and were resilient in the short, mid, and long terms (T5, T30, and T92). Denitrifying enzymes originate from an enzyme pool known to be tolerant of extended drought periods, which would explain the rapid recovery of the denitrification process after rewetting (Peterjohn, 1991). A recent experiment on a managed grassland, conducted by Harris et al. (2021), also monitored an increase in N_2_O fluxes that the authors attributed to denitrification when rewetting the soils after a drought period. Davidson (1992) reported that denitrification could be resilient in response to drought stress as it can be activated within minutes to hours after soil rewetting and the flush of available C and N caused by the rewetting of stressed soils can lead to the resuscitation of dormant denitrifiers (Six et al., 2002). Another sign that the denitrifying bacterial communities were affected by stress was the decrease at T0 of the abundances of *narG* and *nosZ* genes in LO1, HO, and LO2. Unlike PDR, the abundances of genes involved in denitrification were not resilient at T5 and T30 following rewetting. Consequently, during extreme dry conditions, we might expect a decrease in denitrification and denitrifying bacterial populations as shown by Bremner and Blackmer (1978) and later by Butterbach-Bahl and Dannenmann (2011). However, in the present study we chose to monitor *narG* and *nosZ* gene copy numbers as indicators of the impact of the heat and drought stress on denitrification, although other genes are also key functional markers of denitrification; in particular, *nirK* and *nirS* genes play an important role in an intermediary step of denitrification, the production of NO which is rapidly further reduced to N_2_O. These two genes are in the genome of nitrite-reducing denitrifiers, which consists of a highly phylogenetically diverse functional guild (Wei et al., 2015; Bonilla-Rosso et al., 2016). Thus, the resilience of PDR in our experiment could be attributed to nitrite-reducing activity, implying that it was the final denitrification step and reduction of N_2_O to N_2_ that was highly impacted by the stress. Further work would enable to specifically distinguish impacts of heat and drought stress on the different steps of the denitrification process.

#### Nitrification Activities Were Resistant, as Were Gene Abundances

PNR showed no significant differences between stressed soils and controls for all soils at T0, suggesting that the nitrifying communities in all soils were resistant to stress. Hartmann et al. (2013) reported similar findings in grassland soils where PNR were not affected by drought in experimental plots where precipitations were blocked for 41 days. Furthermore, nitrification potentials were reported to be higher in dry and hot summer seasons compared to wet seasons in grasslands in California United States (Parker and Schimel, 2011). Drought actually increased the abundance of *amoA* genes in IS soil, which is in accordance with Bremner and Blackmer (1978) and Kaurin et al. (2018), who reported that during extreme conditions an increase in nitrifiers and nitrification is expected. Davidson (1992) also reported drought-resistant nitrifying microbial communities that are adapted to survive long dry summer seasons in grasslands. However, PNR in LO1 and HO decreased significantly 5 days after rewetting (T5), which is in accordance with results reported by Tan et al. (2018) showing that nitrification rates decreased significantly with soil moisture increase, contrarily to denitrification that increases with moisture. However, overall *amoA* gene abundances in soils were lower than abundances reported by other studies in German arable soil and in rice paddy fields (Radl et al., 2015; Zhang et al., 2018, respectively). This may be due to the domination of nitrifying archaeal over bacterial groups especially under drought conditions in arable soils (Radl et al., 2015) and in urban soils (Wang et al., 2017). In contrast, our results were somewhat comparable to mountain abandoned meadow soils (Fuchslueger, 2014). Overall the N-cycling function seemed less affected by stress than the C-cycling and the phosphate dynamics-function as PDR was resilient and PNR was resistant.

#### No Significant Correlations Were Found Between Soil N-Cycling Processes and Functional Genes Abundances

In the present study, we found no significant correlations between *narG* and *nosZ* functional genes abundances and corresponding N-cycling functions (data not shown). Although some studies have found that denitrifier and nitrifier community compositions are important factors in the regulation of denitrification and nitrification processes (Petersen et al., 2012; Zhang et al., 2017, 2018; Zhang and Ji, 2018), others did not suggest any relationship between the two (Hallin et al., 2009; Attard et al., 2011; Graham et al., 2014). One likely reason for these differences is that many microorganisms carry denitrification genes at any given moment but only a subset of the total community is active and carries out denitrification. Another reason for the lack of a relationship in the present work could be the interference of other parameters such as abiotic characteristics, environmental conditions, or the lack of data to show a clear trend. In a study conducted by Graham et al. (2016), the authors suggested that N-cycling processes are more driven by the edaphic characteristics of soils than by the abundances of functional genes in tropical rainforest soil samples using only edaphic factors. In addition, models containing only edaphic factors data explained N-cycling variation more than models using both gene abundance data. Rocca et al. (2015) published a review where they conducted a meta-analysis of the relationships between gene abundances and corresponding process rates linked to the C- or N-cycling. Authors reported that within the 59 chosen studies, there was a significant but weak positive relationship between gene abundance and the corresponding process (*r* = 0.3, *p*< 0.0001, *n* = 189) using the Pearson product–moment test. They analyzed studies exclusively using gene abundances measured by qPCR and including the bacterial *amoA* gene in relation to nitrification, and *narG* and *nosZ* genes to denitrification. Thus, the non-significant correlations found in our study might also be due to dataset dimensions. Again, further analysis of other functional genes encoding different steps of nitrogen cycling, such as *nirK* and *nirS* as mentioned previously, could bring more insight on the links between activity measurements and potentials.

## Conclusion—The Pros and Cons of Soil Rehabilitation on Carbon Mineralization and Nitrogen Cycling

Heat and drought stress affected both carbon mineralization and phosphate dynamics by altering soil respiration and microbial enzymatic activities, in some cases causing irreversible shifts in the activity’s state. This is probably due to stress causing cell desiccation, lysis, and thermal denaturation of enzymes. Soil respiration showed resilience after 96 days, possibly due to desiccation-resistant bacteria such as *Actinobacteria* or *Firmicutes*. Furthermore, the non-rehabilitated soil IS showed resistance of microbial activity involved in nutrient cycling functions, suggesting a previous adaptation of its microbial communities to drought and heat stress as the soil was not rehabilitated. Thus, it can be suggested that an initial soil could be more resistant and resilient in terms of soil functioning as its microbial communities may have acquired a greater flexibility to environmental conditions. However, this soil is characterized with a low level of activity compared to other soils, which highlights the importance of organic matter contents in stimulating microbial activities. Furthermore, microbial activities involved in carbon cycling and nutrient availability in HO were more resilient to stress than LO1 and LO2, highlighting this rehabilitation trajectory as the most optimal on the short term. Our findings highlight the importance of organic matter content and pre-exposition to stress in determining the performance of soil carbon cycling and phosphate dynamics in rehabilitated urban soils.

Microbial activities and genetic potentials regarding processes involved in nitrogen cycling were more resilient. Denitrification processes were sensitive to heat and drought as they decreased significantly in all soils following the stress. However, they recovered quickly due to the resilience of denitrification enzymes and their tolerance to drought. Furthermore, denitrifying bacterial communities were also affected by stress as *narG* and *nosZ* gene abundances decreased significantly but did not recover after rewetting, suggesting that rewetting soils either boosts denitrifying communities’ activity, not abundance, or boosts nitrite-reducing denitrifiers increasing N_2_O production but not N_2_. Further measurements of *nirK* and *nirS* gene abundance, which encode nitrite reduction, are required to elucidate this. In contrast, PNR were not affected by stress, and nitrifying bacterial communities showed resistance to stress as *amoA* gene abundances were not affected. Moreover, no correlations were found between functional gene abundances and corresponding denitrification and nitrification rates, which is probably due to the interference of other environmental parameters or simply because of our dataset dimensions.

In conclusion, although the poor initial state of the existing soil IS was confirmed by the low activities we measured, it was resistant and resilient to the heat and drought stress, probably due to its pre-exposure to these stress *in situ*. Among the rehabilitation solutions tested in the present study, i.e., either an organic-matter-rich technosol (HO) or a less rich organic-matter technosol (LO1 and LO2), although the LO soils were the cheaper option, they suffered more from the heat and drought stress, suggesting that even though the initial cost was more important for HO, it may be the better solution on the long run. Also, considering the LO1 and LO2 soils, it appeared that globally in the control soils during the experiment, LO1 activities and gene abundances were overall higher than in LO2 control soils. Future work is required to understand the impact of the types of plants seeded on site on these differences. Finally, an in-between solution may be to amend the IS with organic matter or adapted seeds and microbial inoculation as the bacterial communities in this soil are already resistant and resilient to temperature and drought stress.

## Data Availability Statement

The raw data supporting the conclusions of this article will be made available by the authors, without undue reservation.

## Author Contributions

MF, CJ, MM-H, M-PN, and JH: conceptualization, methodology, validation, writing—review and editing, and supervision. MF and M-PN: experiment, sampling, microbiological analysis, and investigation. MF: writing original draft preparation and data curation. CJ, MM-H, and JH: project administration and funding acquisition. All authors have read and agreed to the published version of the manuscript.

## Conflict of Interest

The authors declare that the research was conducted in the absence of any commercial or financial relationships that could be construed as a potential conflict of interest.

## Publisher’s Note

All claims expressed in this article are solely those of the authors and do not necessarily represent those of their affiliated organizations, or those of the publisher, the editors and the reviewers. Any product that may be evaluated in this article, or claim that may be made by its manufacturer, is not guaranteed or endorsed by the publisher.

## Footnotes

1
www.valorhiz.fr

2
https://www.microresp.com/
