# Supplementary material for: Resistance and Resilience of Soil Nitrogen Cycling to Drought and Heat Stress in Rehabilitated Urban Soils
Source: Front Microbiol. 2021 Dec 22;12:727468. doi: 10.3389/fmicb.2021.727468 (PMC8727462; doi:10.3389/fmicb.2021.727468)
Supplement: Supplementary file 1 [file Data_Sheet_1.PDF]

Tableau SM1: Means of 16S rRNA gene abundances for Initial Soil (IS), Low Organic matter soil 1 (LO1), Low Organic matter soil 2 (LO2) and High Organic matter soil (HO) and their controls TIS, TLO1, TLO2 and THO at different sampling times (T0, T5, T30 and T92). Statistical tests were performed using Kruskal-Wallis test ($p < 0.05$). The asterisks indicate a significant difference between a stressed soil and its control at the same sampling time. Error bars represent the error of means.

samples	TIMES	16S (copies $g^{-1}_{dry\ soil}$)	ET
TIS	T0	$1.3x10^8$ *	$3.2x10^7$
IS	T0	$2.7x10^8$	$1.0x10^7$
TLO1	T0	$2.5x10^8$ *	$6.5x10^7$
LO1	T0	$3.7x10^8$	$4.4x10^7$
TLO2	T0	$1.5x10^8$ *	$2.9x10^7$
LO2	T0	$4x10^8$	$6.0x10^7$
THO	T0	$2.7x10^8$ *	$3.8x10^7$
HO	T0	$3.9x10^8$	$1.0x10^8$
TIS	T5	$1.0x10^8$ *	$2.3x10^7$
IS	T5	$6.2x10^7$	$1.1x10^7$
TLO1	T5	$3.2x10^8$ *	$3.7x10^7$
LO1	T5	$9.9x10^7$	$3.2x10^7$
TLO2	T5	$6.4x10^7$ *	$1.4x10^7$
LO2	T5	$2.2x10^8$	$7.0x10^7$
THO	T5	$1.9x10^8$ *	$5.3x10^7$
HO	T5	$9.3x10^7$	$7.6x10^6$
TIS	T30	$8.4x10^7$ *	$8.6x10^6$
IS	T30	$4.3x10^7$	$1.8x10^7$
TLO1	T30	$1.4x10^8$ *	$1.4x10^7$
LO1	T30	$8.9x10^7$	$2.9x10^7$
TLO2	T30	$1.1x10^8$ *	$2.3x10^7$
LO2	T30	$3.1x10^7$	$1.0x10^7$
THO	T30	$2.0x10^8$	$2.4x10^7$
HO	T30	$1.1x10^8$	$1.2x10^7$
TIS	T92	$1.3x10^8$ *	$5.4x10^6$
IS	T92	$6.2x10^7$	$2.1x10^7$
TLO1	T92	$1.5x10^8$	$2.6x10^7$
LO1	T92	$1.1x10^8$	$1.2x10^7$
TLO2	T92	$7.3x10^7$	$3.2x10^7$
LO2	T92	$7.0x10^7$	$1.7x10^7$
THO	T92	$1.3x10^8$	$4.3x10^6$
HO	T92	$1.2x10^8$	$1.3x10^7$
