# Supplementary material for: Resistance and Resilience of Soil Nitrogen Cycling to Drought and Heat Stress in Rehabilitated Urban Soils
Source: Front Microbiol. 2021 Dec 22;12:727468. doi: 10.3389/fmicb.2021.727468 (PMC8727462; doi:10.3389/fmicb.2021.727468)
Supplement: Supplementary file 2 [file Data_Sheet_2.PDF]

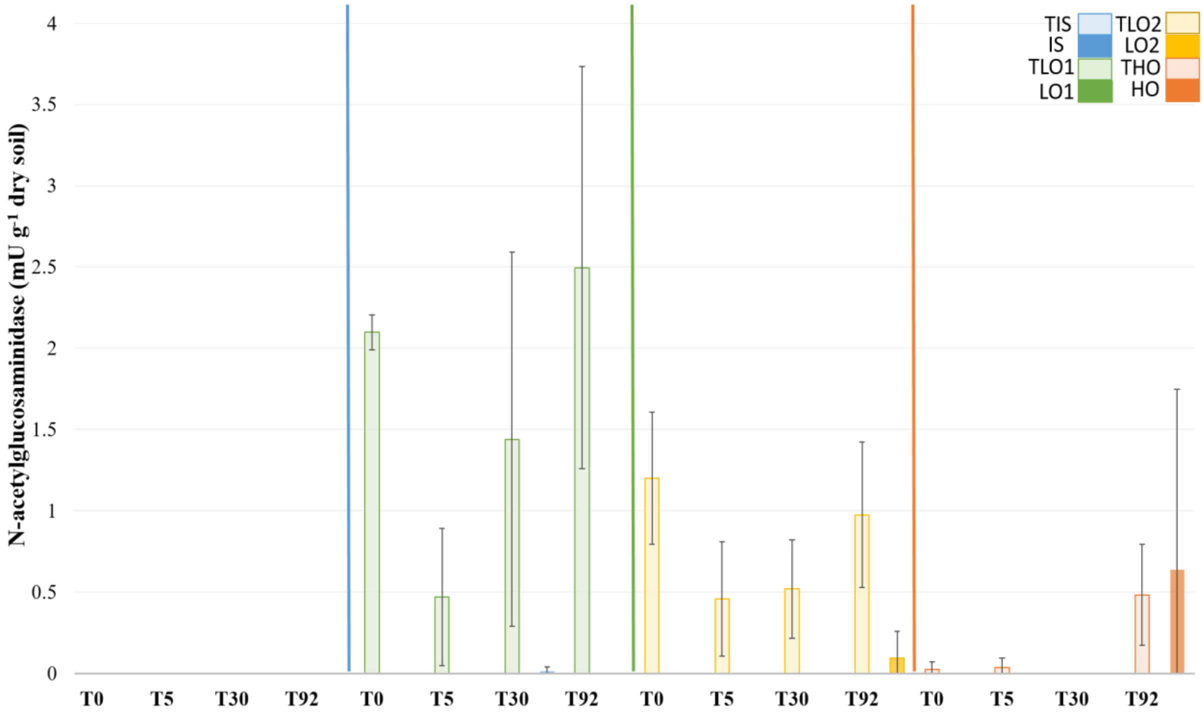

Figure SM1: Means of N-acetylglucosaminidase for Initial Soil (IS), Low Organic matter soil 1 (LO1), Low Organic matter soil 2 (LO2) and High Organic matter soil (HO) and their controls TIS, TLO1, TLO2 and THO at different sampling times (T0, T5, T30 and T92). Statistical tests were performed using Kruskal-Wallis test ($p < 0.05$).
